# Rapid rise of the ESBL and *mcr-1* genes in *Escherichia coli* of chicken origin in China, 2008–2014

**DOI:** 10.1038/s41426-018-0033-1

**Published:** 2018-03-14

**Authors:** Congming Wu, Yingchao Wang, Xiaomin Shi, Shuang Wang, Hongwei Ren, Zhangqi Shen, Yang Wang, Juchun Lin, Shaolin Wang

**Affiliations:** 1 Beijing Advance Innovation Center for Nutrition and Human Health, College of Veterinary Medicine, China Agricultural University, Beijing, 100193 China; 2 College of Veterinary Medicine, Sichuan Agricultural University, Wenjiang, 611130 China

## Abstract

Extended-spectrum beta-lactamase-producing *Escherichia coli* (ESBL-EC) strains are emerging around the world as a source of resistance to β-lactam antibiotics such as ampicillin, cefotaxime, and ceftazidime. *mcr-1* is a novel plasmid-mediated gene conferring resistance to colistin. The aim of this study was to investigate the prevalence of ESBL-EC *mcr-1* of chicken origin in the different provinces of China during 2008–2014. Overall, 341 of 821 isolates were determined to be ESBL-EC strains, and the proportion of ESBL-positive strains almost doubled from 2008 to 2014. The findings of our study revealed regional differences, with significantly more ESBL-EC isolates from stockbreeding in concentrated poultry industry areas in Shandong than from the other four provinces. The ESBL type analysis showed that *bla*_CTX-M_ was the most prevalent ESBL-encoding gene (92.7%). In total, twelve subtypes of CTX-M genes were detected, among which, *bla*_CTX-M-55_ (34.3%) and *bla*_CTX-M-65_ (17.9%) were the major identified genotypes. In addition, *bla*_TEM_ and pAmpC genes were carried by 86.0% and 8.5% of isolates, respectively. In this study, we also observed 44 *E. coli* isolates with multiple ST types (ST46, ST1286, ST10, ST29, ST101, and ST354) carrying *mcr-1*, and the majority of *mcr-1*–carrying plasmids were IncI2. The whole-genome sequencing analysis indicated the co-existence of *bla*_CTX-M_ and *mcr-1* in ESBL-EC of both animal and human origin, and phylogenetic analysis further revealed their close relationship, especially several isolates sharing a small number of SNPs, which suggested the increasing trend of co-existence and transmission of ESBL and *mcr-1* in both clinical medicine and veterinary medicine.

## Introduction

Antibiotic resistance in bacteria has become one of the major threats to public health because of the extensive use of antibiotics in human medicine and animal farming. Global antimicrobial consumption in food animal production may increase by 67% by 2030, driven primarily by BRICS (Brazil, Russia, India, China, and South Africa) countries, as large-scale and intensive farming operations are heavily in demand with the rise in income and meat consumption^1^. One study predicted that antimicrobial consumption in chickens and pigs in Asia would grow by 129% and 124%, respectively, and the remarkable growth in consumption of chickens is primarily a result of the rapid expansion of the breeding industry in India^2^. Over 50% of total antibiotic production is used routinely in subtherapeutic doses for growth promotion, and this corresponds to a consumption of antimicrobials per kilogram of animal produced of ~148 mg kg^−1^ in chicken production^3^. The rapid increase in the multi-drug resistance of *E. coli* has been observed not only in clinical medicine^4^, but also widely in food animal production^5^, particularly with an increasing prevalence of ESBL and AmpC β-lactamase producing strains, which greatly compromises the therapeutic efficacy and increases morbidity and mortality.

ESBLs are enzymes that confer resistance to most β-lactam antibiotics, especially to third-generation cephalosporins (e.g., ceftazidime, cefotaxime, and ceftriaxone) and aztreonam but not to cephamycins (cefoxitin and cefotetan) and carbapenems^6,7^. The majority of ESBLs are *bla*_TEM_, *bla*_SHV_ and *bla*_CTX-M_ types and CTX-M-producing *E. coli* isolates, which are recognized as a major cause of hospital and community-onset infections^8–10^. ESBL-EC is usually considered an indicator bacterium to trace the spread of antibiotic resistance genes, as resistance genes transfer between the same species and different species through genetic elements, especially plasmids^11^. Plasmid-encoded AmpC (pAmpC) β-lactamases or ESBL conferring resistance to penicillins; first, second, and third-generation cephalosporins; and monobactams have been reported both in human and food animal isolates worldwide. CMY-2 is the most common pAmpC in *E. coli* in several geographical areas, including Asia, North America, and Europe^12–15^, and has been reported in food animals on all continents except Australia^16,17^. ESBL and pAmpC genes have been widely detected in food-producing animals, especially in poultry and retail meat^18–20^.

Moreover, *mcr-1*, a novel colistin-resistance mechanism, was discovered in 2015 and caught world-wide attention^21^. Multiple studies have confirmed its wide spread in both clinical settings^22^ and animal husbandry^23^. After the discovery of *mcr-1*, the Chinese government implemented a fast track and banned the use of colistin as a growth promoter, effective since April 1st, 2017, and the European Medicines Agency immediately initiated the re-assessment of colistin use in food animal production and advised member countries to reduce the usage of colistin in these animals^24^. However, the emergence and prevalence of *mcr-1* are still not well understood. Although one specific study revealed that the emerging and elevated spreading of *mcr-1* in food animals could be traced back to 2009, details are still lacking. The elevated prevalence of ESBL has been observed since 2009, as revealed in previous studies, and the coexistence of ESBL and *mcr-1* in an *E. coli* strain was first reported in China in 2016^25^. A recent study suggested that cephalosporin resistance genes are mainly disseminated in animals and humans via distinct plasmids^26^. It is highly likely that food animals have become one of the most important sources for the spread of these resistance-gene-carrying bacteria to humans through horizontal gene transfer. To evaluate the co-existence and prevalence of ESBL and *mcr-1* in *E. coli* of chicken origin, we investigated the trends of ESBL-EC prevalence in chickens in the different provinces of China from 2008 to 2014 and further elucidated the predominant genotype of ESBL and the phylogenetic relationship among ESBL-EC carrying *mcr-1*.

## Results

### Prevalence of ESBL-EC

**Fig. 1:**
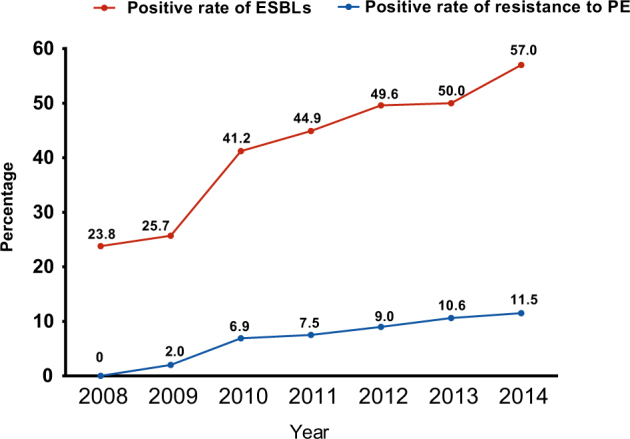
**Positive rate of ESBL and polymyxin E resistance from 2008 to 2014**

**Fig. 2:**
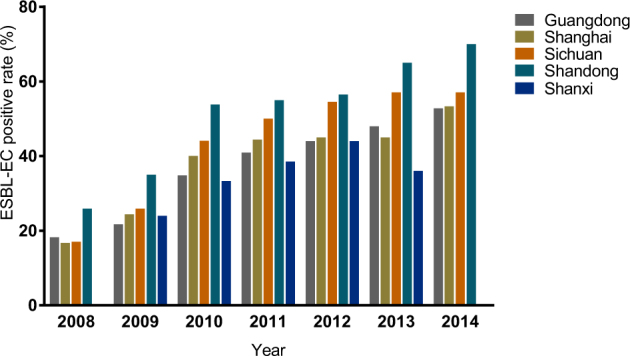
**Positive rate of ESBL-EC in five broiler production regions of China from 2008 to 2014**

**Fig. 3:**
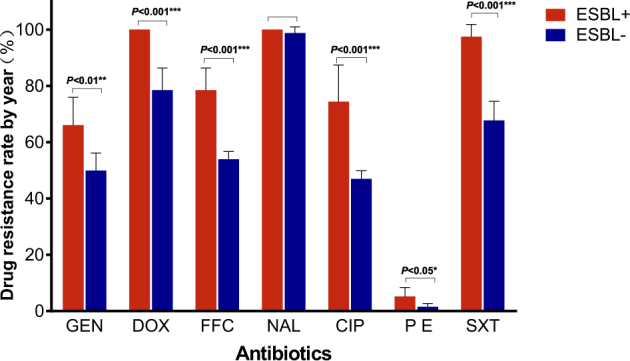
**Multiple drug resistance rate between ESBL-EC and non-ESBL-EC**

We obtained 341 ESBL-EC strains from 821 isolates of chicken origin (Table 1), and the ESBL-EC increased from 23.8% in 2008 to 57.0% in 2014 (Fig. 1). Although there were slight differences in the resistance rates among the five sampling regions, they all showed elevated resistance from 2008 to 2014 (Table 1 and Fig. 1). Shandong Province had the highest rate, while the lowest rate was in Shanxi Province (Fig. 2). The ESBL-EC also showed considerable multiple drug resistance to various non-β-lactamase antibiotics, including doxycycline, ciprofloxacin, gentamicin, nalidixic acid, sulfamethoxazole, and florfenicol (Table 2). The resistances to tetracycline, nalidixic acid, and sulfamethoxazole were 100% in ESBL-EC isolates. Multiple drug resistance of ESBL-positive strains was significantly higher than that of ESBL-negative isolates for 6 major antibiotic drug classes, except nalidixic acid, as the resistance of both ESBL-EC positive and negative strains peaked (Table 2 and Fig. 3).

**Table 1:** No. of strains, ESBL-EC strains and ESBL-EC positive rates across different provinces from 2008 to 2014

**Year**	**Province**			**Total**		
	Guangdong	Shanghai	Shanxi	Sichuan	Shandong	
**2008**	22^a^(4^b^,18.2^c^)	12(2,16.7)	–	58(15, 25.9)	13(4, 30.8)	105^d^(25^e^, 23.8^f^)
**2009**	23(5, 21.7)	45(11, 24.4)	25(6, 24.0)	27(7, 25.9)	20(7,35)	140(36, 25.7)
**2010**	23(8, 34.8)	20(8, 40)	12(4, 33.3)	34(15, 44.1)	13(7, 53.8)	102(42, 41.2)
**2011**	22(9, 40.9)	18(8, 44.4)	39(15, 38.5)	28(14, 50.0)	20(11, 55.0)	127(57, 44.9)
**2012**	25(11,44.0)	20(9, 45.0)	25(11, 44.0)	23(12, 54.5)	22(14, 56.5)	115(57, 49.6)
**2013**	25(12, 48.0)	20(9, 45.0)	25(9, 36.0)	28(16, 57.1)	20(13, 65.0)	118(59, 50.0)
**2014**	36(19, 52.8)	30(16, 53.3)	–	28(16, 57.1)	20(14, 70.0)	114(65, 57.0)
**Total**	176^g^	165	126	226	128	821(341)

^a^ Number of strains

^b^ Number of positive strains

^c^ ESBL-EC positive rate

^d, g^ Total number of strains

^e^ Total number of positive strains

^f^ Total positive rate of ESBL-EC

**Table 2:** Elevated multiple drug resistance rate in the *E. coli* of chicken origin from 2008 to 2014

Antibiotics	Species	Drug resistance rate by year (%)						
		2008	2009	2010	2011	2012	2013	2014
GEN	ESBL^+^	52.9	54.8	63.0	65.7	72.2	76.04	77.8
	ESBL^−^	41.9	42.3	48.7	50.3	52.9	56.7	56.9
DOX	ESBL^+^	100	100	100	100	100	100	100
	ESBL^−^	67.6	71.4	74.1	78.9	83.3	85.3	88.9
FFC	ESBL^+^	67.6	71.4	74.1	78.9	83.3	85.3	88.9
	ESBL^−^	50.3	51.4	53.3	53.4	54.9	56.1	58.4
NAL	ESBL^+^	100	100	100	100	100	100	100
	ESBL^−^	93.9	98.6	98.7	100	100	100	100
CIP	ESBL^+^	52.9	59.2	75.9	80.3	81.9	84.0	86.4
	ESBL^−^	43.6	45.0	45.3	47.1	46.4	48.4	52.6
P E	ESBL^+^	0.0	2.0	5.6	5.6	6.9	8.0	8.6
	ESBL^−^	0.0	0.0	1.3	1.9	2.1	2.6	2.9
SXT	ESBL^+^	88.2	96.0	98.1	100	100	100	100
	ESBL^−^	57.0	60.5	68.0	68.3	71.2	73.2	75.9

*GEN* gentamicin, *DOX* doxycycline, *FFC* florfenicol, *NAL* nalidixic acid, *CIP* ciprofloxacin, *PE* polymyxin E, *SXT* compound sulfamethoxazole

### Characterization of the ESBLs genes in the *E. coli* isolates

**Fig. 4:**
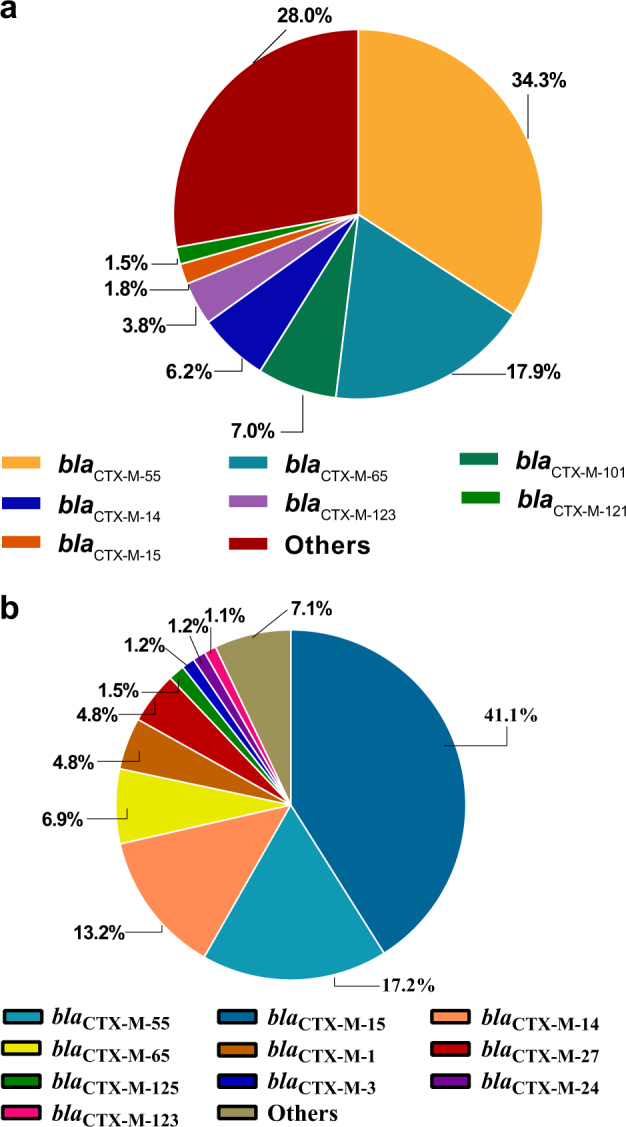
The distribution of CTX-M types of ESBL-EC. **a** The distribution of CTX-M types of ESBL-EC of chicken origin in China. **b** The distribution of CTX-M types of ESBL-EC from the NCBI whole-genome sequencing database

In this study, 92.7% (316/341) of ESBL-EC isolates contained *bla*_CTX-M_ genes, among which, *bla*_CTX-M-55_ (34.3%), *bla*_CTX-M-65_ (17.9%), and *bla*_CTX-M-101_ (7.0%) were the most dominant *bla*_CTX-M_ types (Fig. 4a)^27–32^. To the best of our knowledge, for the first time, we identified *bla*_CTX-M-98_ in *E. coli* of food animals in China, which has been detected in humans previously^33,34^. We also analyzed over 6000 whole genome sequences of *E. coli* isolates submitted to the NCBI database. The distribution of all CTX-M-producing *E. coli* isolates (*n* = 660) from the NCBI database were also analyzed and presented (Fig. 4b). As over 80% of them were from human isolates, *bla*_CTX-M-15_ (41.1%) was the dominant type in the human isolates.

Of the isolates from this study, 86.0% were detected as harboring *bla*_TEM_ genes at a proportion >46.5%; *bla*_TEM-1_ accounted for 80.0% of all *bla*_TEM_ genes, a remarkable prevalence in food animals, and the remaining were *bla*_TEM-52_ and *bla*_TEM-116_ genes. pAmpC encoding genes, which were found in 37 tested strains, included CMY2 (19) and OXA10 (18) but not SHV, as previously reported^11,16,34^. ESBL or AmpC-producing *E. coli* were further investigated for genetic relationship using PFGE (Supplementary Figure S1). The results illustrated that most of the strains isolated in this study were clonally unrelated, similar to previous reports^35^.

### Co-existence of ESBL and *mcr-1* genes and whole-genome sequencing analysis

From this study, the emergence of resistance to polymyxins has also been observed since 2009, and it has been increasing in the last few years (Table 1 and Fig. 1). To further understand the co-existence of *mcr-1* and ESBL, all colistin-resistant isolates were selected for the screening of *mcr-1* and other *mcr* genes (*mcr-2, mcr-3, mcr-4*, and *mcr-5*) using a PCR method. Only *mcr-1* was identified; none of other *mcr* genes was identified. All 44 *mcr-1*–carrying isolates were analyzed using whole genome sequencing to retrieve the complete antibiotic resistance gene profiles. Through the whole genome sequencing, multiple MLST types have been identified, such as ST46 (9), ST1286 (4), ST10 (3), ST29 (3), ST101 (3), and ST354 (3) (Table 3). Furthermore, information on plasmids carrying *mcr-1* was also revealed (Table 3). The Inc type of the majority of these *mcr-1* carrying plasmids was IncI2, and the plasmid size ranged from 58 to 62 kb. Several *mcr-1–*carrying contigs were not sufficiently long to identify the plasmid size and Inc type. The insert sequence ISApl1 surrounding *mcr-1* was identified in the most IncI2 plasmids. Two isolates were identified as carrying Tn6330 (R26 and R46). The whole-genome sequence data revealed that 77.3% of *mcr-1* positive isolates (34/44) carried at least one ESBL gene, including *bla*_CTX-M_ (*n* = 25), *bla*_OXA_ (*n* = 11), and *bla*_TEM_ (*n* = 25). *bla*_TEM-198_ (7/25) is the dominant type in the *bla*_TEM-1_, and *bla*_CTX-M-55_ (6/44), *bla*_CTX-M-14_ (6/44), *bla*_CTX-M-65_ (4/44) are the three major *bla*_CTX-M_ types. Eleven isolates carry both *bla*_CTX-M_ and *bla*_TEM_ genes; one isolate carries *bla*_CTX-M-15_, *bla*_CTX-M-65_, *bla*_OXA-1_, *bla*_OXA-10_, and *bla*_TEM-141_; and six isolates contain multiple ESBL genes (*bla*_TEM_-*bla*_CMY_-*bla*_OXA,_ *bla*_TEM_- *bla*_CTX-M_-*bla*_OXA,_ *bla*_TEM_-*bla*_CTX-M_-*bla*_CMY_).

**Table 3:** The information regarding *mcr-1* positive *E. coli* and *mcr-1–*carrying plasmids

*E. coli* Isolate ID	BioSample ID	Year	Area	MLST Type	Plasmid Inc type	*mcr-1* contig size (bp)	No. of contigs
R02	SAMN07983251	2011	Chongqing	10	−	2943	74
R24	SAMN07983271	2011	Sichuan	10	IncI2	60,207	28
R46	SAMN07983287	2010	Shanghai	10	IncI2	61,831	21
R26	SAMN07983273	2013	Shanghai	29	IncI2	60,873	31
R37	SAMN07983280	2010	Guangzhou	29	IncI2	60,955	32
R95	SAMN07983324	2013	Shanghai	29	IncI2	60,178	34
R10	SAMN07983257	2013	Shanghai	46	IncI2	59,879	27
R12	SAMN07983261	2012	Shanghai	46	IncI2	61,943	25
R13	SAMN07983262	2013	Shanghai	46	IncI2	62,380	25
R19	SAMN07983267	2013	Shanghai	46	IncI2	61,022	27
R35	SAMN07983279	2012	Shanghai	46	–	11,027	84
R40	SAMN07983283	2012	Shanghai	46	IncI2	58,887	29
R53	SAMN07983292	2013	Shanghai	46	IncI2	63,724	26
R56	SAMN07983295	2012	Shanghai	46	IncI2	60,445	28
R64	SAMN07983303	2010	Shanghai	46	IncI2	61,022	27
R34	SAMN07983278	2011	Chongqing	48	IncI2	60,203	32
R59	SAMN07983298	2012	Guangzhou	93	–	2863	164
R61	SAMN07983300	2013	Shandong	93	IncI2	60,872	27
R14	SAMN07983263	2014	Sichuan	101	IncI2	60,872	35
R28	SAMN07983275	2011	Guangzhou	101	IncI2	60,872	34
R41	SAMN07983284	2012	Sichuan	101	IncHI2A + IncHI2 + IncI2	257,133	6
R44	SAMN07983286	2014	Shandong	156	IncI2	58,932	28
R47	SAMN07983288	2014	Sichuan	165	IncI2	62,807	26
R07	SAMN07983254	2011	Shanghai	354	IncI2	59,541	28
R48	SAMN07983289	2011	Shanghai	354	–	9910	65
R51	SAMN07983291	2011	Shanghai	354	–	20,901	46
R75	SAMN07983308	2011	Shanghai	354	IncI2	59,294	29
R09	SAMN07983256	2009	Guangzhou	533	−	11,573	43
R57	SAMN07983296	2009	Guangzhou	533	−	11,525	45
R74	SAMN07983307	2009	Guangzhou	533	−	11,022	44
R25	SAMN07983272	2013	Shanghai	542	−	2943	94
R39	SAMN07983282	2013	Shanghai	542	−	2943	90
R38	SAMN07983281	2014	Shandong	617	IncI2	61,088	25
R27	SAMN07983274	2014	Shandong	648	IncI2	60,752	24
R01	SAMN07983250	2012	Unknown	1286	IncI2	61,068	27
R134	SAMN08180604	2012	Shanghai	1286	–	11,029	49
R62	SAMN07983301	2014	Sichuan	1286	IncI2	64,482	29
R78	SAMN07983311	2012	Shanghai	1286	–	12,112	45
R86	SAMN07983317	2012	Shanghai	1286	IncI2	61,931	27
R96	SAMN07983325	2012	Shanghai	1286	–	10,915	48
R50	SAMN07983290	2010	Shanghai	1564	–	2946	82
R58	SAMN07983297	2012	Shanghai	1589	IncI2	62,044	29
R31	SAMN07983276	2012	Sichuan	3014	IncI2	60,874	27
R32	SAMN08238410	2014	Shandong	5229	–	4631	67

**Fig. 5:**
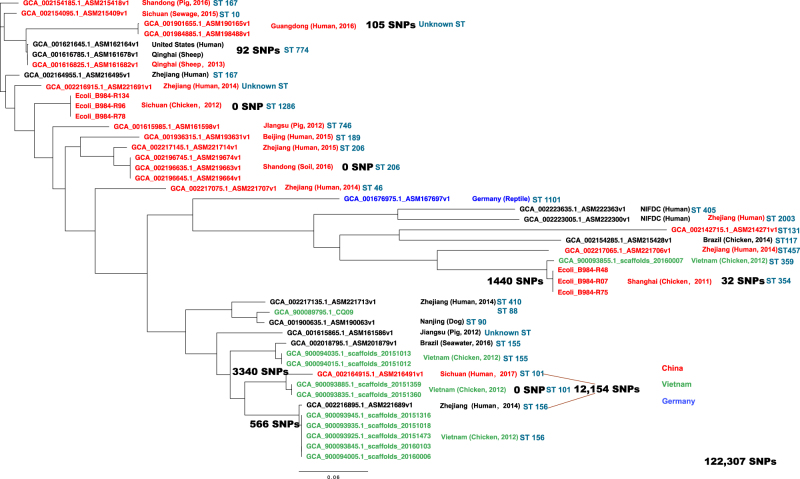
The phylogenetic tree of ESBL-EC carrying both *bla*_CTX-M-55_ and *mcr-1*. * Red, ESBL-EC from China; Green, ESBL-EC from Vietnam; Blue, ESBL-EC from Germany

In total 39 *bla*_CTX-M-55_–carrying isolates of different origin from four countries (China, Germany, United States, Vietnam) from the NCBI database were identified, combined with 6 ESBL-EC isolates carrying both *bla*_CTX-M-55_ and *mcr-1* from this study and subjected to a core genome phylogenetic analysis (Fig. 5). In total 3 of 6 isolates from this study had *bla*_CTX-M-55_ surrounded by ISEcp1. A total of 122,307 SNPs were used to calculate the phylogenetic relationship among these isolates; the number of SNPs among closely related isolates ranged from 0 to 105 SNPs. Interestingly, 34 of 45 isolates were identified as carrying both *bla*_CTX-M-55_ and *mcr-1*, and ESBL-EC of animal origin had a higher positive rate (83.3%, 25 of 30) compared with human origin (60%, 9 of 15). Several isolates carrying both *bla*_CTX-M-55_ and *mcr-1* from animal and human origin were clustered very closely and belonged to geographically distinct regions, e.g., China and Vietnam.

## Discussion

China has the second largest broiler production and the largest consumption of antibiotics in the world^36^, and the overall antibiotic consumption has continued rising, which could potentially facilitate the spread of antibiotic resistance. The prevalence of ESBL-EC of chicken origin in China has been increasing since 2006, with slight differences among geographical locations and animal species^5^. Recently, *mcr-1*, *mcr-2*, and *mcr-3*, a group of genes mediating colistin resistance through plasmids, have been widely discovered around the world^21,37,38^. The mechanism of colistin resistance related to *mcr-1* and *mcr-2* has also been revealed^39,40^. However, there are few reports studying the prevalence of the co-existence of *mcr-1* and ESBL in the broiler production. Herein, we screened over 800 *E. coli* isolates from several major broiler production areas of China during 2008-2014 and found that the fast rise of ESBL and emergence of *mcr-1–*positive EC (MCRPEC) in the chicken were consistent with previous reports^5,41^. This investigation is the first to reveal the co-rising of ESBL and *mcr-1* in *E. coli* isolates in a longitudinal study.

In this study, the positive rate of ESBL-EC of chicken origin in China was higher than that in some previous reports^28–30,32,42^ but lower than that in other reports^6,28,43,44^ for the same period, in which the sampling area and total number of isolates were relatively limited and did not cover major poultry production areas. This study surveyed three major broiler production areas, namely, Guangdong, Shandong, and Sichuan Provinces, accounting for ~40% of the total broiler production in China^45^, which should give a more comprehensive estimation of major poultry production areas compared with previous studies. One report from PNAS also confirmed that an antibiotic consumption per 10 km^2^ in chickens exceeding 250 kg in these areas has the highest density throughout China^2^. Although the positive rate of ESBL-EC differed among geographical locations, the resistance rates all doubled in a short period of time (Table 2 and Fig. 2) and have continued rising steadily. Shandong Province ranks first in poultry production in China; the province’s annual poultry production, including chickens and ducks, has reached 1.87 billion. The highly concentrated poultry industry in Shandong Province may also facilitate the expansion of ESBLs, causing this province to have the highest ESBL rate. These results were similar to those obtained in previous investigations^9,12,28,46^. A very recent study reported that the overall prevalence of ESBLs from India in the broiler chicken was 87.0%, which was much higher than that in China (57.0%, 2014)^1^. In this study, not only has the resistance rate of ESBL-EC strains been increasing at an alarming rate but also ESBL-EC has shown significant resistance to other major antibiotics, such as doxycycline, nalidixic acid, and sulfamethoxazole, with the resistance even reaching 100% in several classes of antibiotics. This was also observed in an ESBL study of India chicken production with overwhelming multiple drug resistance to nalidixic acid, ciprofloxacin, and chloramphenicol^1^. Such phenomena may be attributed to the co-selection of antibiotic resistance caused by improper combination usage of multiple classes of broad-spectrum antibiotics. The presence of these multiple drug resistance phenotypes further indicated that the co-existence of ESBLs with other resistance genes in *E. coli* strains results in an expansion of their resistance spectrum to β-lactam antibiotics or to other antibiotics, which poses a serious challenge to the application of antibiotics in the poultry industry. Therefore, ESBL-EC may be used as an indicator for the surveillance of multiple-drug-resistant *E. coli* in food animal production.

*bla*_CTX-M_ type was the dominant ESBL-encoding gene in the ESBL-EC^6,11,28,30,32^. Of the 341 ESBL-EC isolates in this study, 92.7% had *bla*_CTX-M_ genes, slightly higher than the previously reported 86% in chickens in China^6,11^ but lower than those (96.9%) in human isolates^47^. The predominant *bla*_CTX-M_ genotype, *bla*_CTX-M-55_, was consistent with the previous report^5,48–51^. Furthermore, the prevalence of *bla*_CTX-M-55_ was even higher than those of *bla*_CTX-M-14_ and *bla*_CTX-M-65_ combined, and the second-most dominant *bla*_CTX-M_ was *bla*_CTX-M-65_, which has surpassed *bla*_CTX-M-14_, compared with previous reports^5^. Overall, the *bla*_CTX-M-1_ group (47%) has replaced the *bla*_CTX-M-9_ (26%) group as the dominant *bla*_CTX-M_ genotype. *bla*_CTX-M-55_ was not reported in human clinical bacteria in China before 2010^33^, but several studies have recently reported the emergence of *bla*_CTX-M-55_ in human isolates, which has become the second-most common *bla*_CTX-M_ enzyme in China, surpassing *bla*_CTX-M-55_^28^.

Interestingly, the prevalence of *mcr-1* was higher in the ESBL-EC than in the non-ESBL-EC (*p* < 0.001, 77.3% vs 22.7%), and the fast rise of ESBL apparently also increased the selective pressure of colistin resistance. Within the *mcr-1* positive *E. coli*, more than 75% of the isolates had at least one ESBL gene, and several isolates carried three or more ESBL genes, suggesting that the ESBL-EC are more likely to recruit the *mcr-1* gene than are non-ESBL-EC. Both β-lactams and colistin could damage bacteria cell walls by inhibiting the synthesis of peptidoglycan and disrupting the outer membrane, respectively. Maintaining the cell wall integrity has become the top priority for bacteria to survive in the battle against antibiotics, leading to the high prevalence of *mcr-1* in ESBL-EC isolates. Recently, from the whole-genome sequence data of ESBL-EC submitted to the NCBI database, over 75% of isolates carried *mcr-1* within the CTX-M-55–positive ESBL-EC, which was much higher than other CTX-M type isolates. According to the whole-genome sequence data of ESBL-EC submitted to the NCBI database, a similar pattern was also observed. Considering that *bla*_CTX-M-55_ has become the dominant *bla*_CTX-M_ type in the ESBL-EC of animal origin in the last decade but was still very rare in the ESBL-EC of human origin; this finding might suggest that *bla*_CTX-M-55_ and *mcr-1* emerged and rose under the heavy selective pressure of antibiotic usage in the animal husbandry in the last decade. Interestingly, one strain isolated from Vietnam in 2012 was clustered together with three other strains isolated in the Shanghai area, all carrying both *bla*_CTX-M-55_ and *mcr-1* and sharing 1440 SNPs (1.2%) of 122,307 total SNPs (Fig. 5). In addition, several ESBL and *mcr-1*-carrying isolates from Vietnam were closely related to the ESBL-positive and *mcr-1–*negative isolates of human origin from Zhejiang Province and shared 566 SNPs. The ST types also confirmed the closely phylogenetic relationship among these isolates (Fig. 5), while a recent study revealed the co-existence of *bla*_CTX-M-15_ and *mcr-1* in the ESBL-EC of human origin^25^. However, these *mcr-1–*negative isolates have the potential capability to eventually acquire the *mcr-1* gene because of its high resemblance of genetic context to the ESBL-EC of animal origin^23,41,52,53^. The whole-genome sequencing also revealed the genetic context of plasmids carrying *mcr-1* in the most *mcr-1*–positive ESBL-EC isolates, which are highly similar to the plasmid pHNSHP45 identified in the previous study^21^. The Inc type of the majority of the *mcr-1–*carrying plasmids was IncI2, which was similar to that of the *E. coli* isolates of pig origin from a recent study^54^. The multiple ST types suggested that the IncI2 plasmid could be adapted to various *E. coli* hosts. Several ST types were the predominant ST types within the prevalence regions, such as ST46, ST354, and ST1286 in Shanghai; however, the same ST type could also be found from different regions, such as ST10. ST354 with an IncI2 plasmid has been reported in the previous study^55,56^.

The co-rising of the ESBL-EC and MECPEC can be traced back to 2008 from this study, which is highly synchronized with the rapid growth of veterinary drug consumption, especially of amoxicillin/clavulanic acid and colistin, in the poultry industry^57^. By 2014, the use of amoxicillin and colistin reached ~7,000,000 kg and 22,000,000 kg, respectively, in food animal production in China^57^. According to the United States FDA, the overall consumption of all penicillin class drugs, including amoxicillin, ampicillin, cloxacillin and penicillin, was only 885,975 kg in 2014^58^. However, from the National Antimicrobial Resistance Monitoring System (NARMS) Integrated Report of 2014, the cephalosporin resistance rate in *E. coli* isolates from retail chicken even declined from a peak of 13% in 2011 to 6.6% in 2014, while the usage of penicillins and cephalosporins increased by 57.0% and 28.0%, respectively^59^. This may suggest that the proper usage of antibiotics during food animal production is crucial to controlling the spread of antibiotic resistance. In the last decade, amoxicillin has been widely used in drinking water for chicks (<7 days) as a preventive measure, and colistin is used as feed additive for the entire production period of chicken. This usage pattern may contribute a significant portion of selective pressure on the bacteria. Although colistin has been banned as a feed additive since April 1st, 2017, it will take some time to observe the decline in colistin resistance in food animals. Taken together, the wide spread of ESBL-EC and *mcr-1* in animal husbandry in China further indicates the extensive use of antibiotics in food animal production, which may facilitate the transmission of antibiotic resistance to humans and the environment and cause a serious threat to health. The phylogenetic analysis also showed the close relationship of ESBL- and *mcr-1*–carrying *E. coli* between the animal isolates and clinical isolates, which suggested that we should pay more attention to monitoring the prevalence of ESBL- and *mcr-1–*carrying *E. coli* in both clinical medicine and food animal production.

## Materials and methods

### Bacterial isolation and antimicrobial susceptibility testing

A total of 821 *E. coli* strains, isolated from cloacal swabbing of chickens, were provided by the China Institute of Veterinary Drug Control. The strains were isolated from different regions between 2008 and 2014 (see Table 1). Screening and confirmation of ESBL producers were conducted according to the guidelines of the Clinical and Laboratory Standards Institute (CLSI, 2013). The minimum inhibitory concentrations (MICs) of cefotaxime or ceftazidime for *E. coli* isolates were initially performed using the broth microdilution method. For isolates with an MIC of cefotaxime or ceftazidime >1 mg/l, the double disk synergy test was further utilized to confirm the ESBL-EC. Seven other antibiotics, namely, doxycycline, gentamicin, ciprofloxacin, compound sulfamethoxazole, polymyxin E, nalidixic acid, and florfenicol, were also tested for MIC using the broth microdilution method. *E. coli* ATCC25922 was used as the quality control strain.

### Detection of the ESBL and pAmpC, and* mcr* genes

ESBL-producing strains were screened for β-lactamase genes, ESBL genes (*bla*_CTX-M_, *bla*_TEM_ and *bla*_SHV_), pAmpC genes (*bla*_CMY_, *bla*_OXA_), and *mcr 1–5* using PCR testing. All the primers used are listed in Table S1. The positive products were sent for Sanger sequencing. The DNA sequences obtained were further checked for ESBL and pAmpC gene subtypes using BLAST analysis (http://www.ncbi.nlm.nih.gov/) or the β-lactamase classification system (http://www.lahey.org/studies/webt.asp).

### Whole genome sequencing analysis and genome typing

All isolates containing *mcr-1* were subjected to whole genome sequencing. Total DNA from the *E. coli* isolates was extracted using a Wizard^®^ Genomic DNA Purification kit (Promega, WI, USA) and then subjected to whole genome sequencing. The library was constructed using a Next^®^ Ultra™ DNA Library Prep kit (New England Biolabs, Ipswich, UK) according to the manufacturer’s protocol, and 250-bp paired-end reads were obtained from an Illumina Hiseq2500 platform (Bionova Biotech Co.). For each *E. coli* isolate analyzed by whole-genome sequencing, at least 100-fold coverage of raw reads were yielded and collected. The clean reads were searched against the ResFinder and PubMLST databases using the SRST2 program to retrieve all the resistance genes and the MLST types as previous study^23^. A draft assembly of the sequences was generated using SPAdes as previous study^23^. A total of 6320 *E. coli* isolate whole-genome sequences (by July 31st, 2017) were download from the NCBI database for the screening of ESBL genes, and all the ESBL-EC isolates were analyzed to retrieve the ESBL genotypes for further analysis. Draft genome sequences were aligned and then applied for phylogenetic tree construction by Parsnp in the Harvest package, and the phylogenetic tree was visualized with FigTree as previously described. Sixty ESBL-EC strains were randomly selected to conduct the PFGE analysis using the methods as described. All whole genome sequences have been submitted to GenBank under BioProject (PRJNA417344).

## Electronic supplementary material

Supplmentary table 1 and figure 1
