# Supplementary material for: Rapid rise of the ESBL and mcr-1 genes in Escherichia coli of chicken origin in China, 2008–2014
Source: Emerg Microbes Infect. 2018 Mar 14;7:30. doi: 10.1038/s41426-018-0033-1 (PMC5849743; doi:10.1038/s41426-018-0033-1)
Supplement: Supplementary file 1 — Supplmentary table 1 and figure 1 [file 41426_2018_33_MOESM1_ESM.docx]

Table S1 PCR Primers

| **Target gene** | **Primer** | **Sequence (50–30)** | **Size of amplicon (bp)** |
| --- | --- | --- | --- |
|  |  |  |  |
| **blaSHV** | bla-SHV.SE | GGTTATGCGTTATATTCGCC | 865 |
|  | bla-SHV.AS | TTAGCGTTGCCAGTGCTC |  |
| **blaTEM** | blaTEM-F | ATGAGTATTCAATTCCG | 861 |
|  | blaTEM-R | CTGACAGTTACCAATGCTTA |  |
| **blaCTX-M-U** | blaCTX-M-U1 | ATGTGCAGYACCAGTAARGTKATGGC | 593 |
|  | blaCTX-M-U2 | TGGGTRAARTARGTSACCAGAAYCAGCGG |  |
| **blaCTX-M-1** | blaCTX-M-1F | AAGACTGGGTGTGGCATTGA | 781 |
|  | blaCTX-M-1R | AGGCTGGGTGAAGTAAGTGA |  |
| **blaCTX-M-9** | blaCTX-M-9F | ATGGTGACAAAGAGAGTGCAACGG | 876 |
|  | blaCTX-M-9R | TCACAGCCCTTCGGCGATGATTCT |  |
| **blaCMY-2** | blaCMY-2F | ATGATGAAAAAATCGTTATGCT | 1145 |
|  | blaCMY-2R | TTATTGCAGCTTTTCAAGAATGCG |  |
| **blaOXA** | blaOXA-1F | AATGGCACCAGATTCAACTT | 599 |
|  | blaOXA-1R | CTTGGCTTTTATGCTTGATG |  |
| **mcr-1** | mcr-1F | ATCAGCCAAACCTATCCTATCG | 1257 |
|  | mcr-1R | ATAGATGTTGCTGTGCGTCTGC |  |
| **mcr-2** | mcr-2F | GCGTAGGCGGTCTAACATGTAT | 378 |
|  | mcr-2R | GCTGACACCTCTTGTCATTGCA |  |
| **mcr-3** | mcr-3F | TATGGGTTACTATTGCTGG | 814 |
|  | mcr-3R | CGATGAGCATCAGGGTAG |  |
| **mcr-4** | mcr-4F | GTCATAGTGGTCGAAAAGTACAG | 667 |
|  | mcr-4R | GTTGGCTCTGATAGACGGTGG |  |
| **mcr-5** | mcr-5F | GCGGTTGTCTGCATTTATCAC | 1042 |
|  | mcr-5R | TGCCGAAGACAGGTTATCAAAG |  |

Fig.S1.The clonality of harboring ESBLs encoding genes strains
